# Supplementary material for: Reconciling Mining with the Conservation of Cave Biodiversity: A Quantitative Baseline to Help Establish Conservation Priorities
Source: PLoS One. 2016 Dec 20;11(12):e0168348. doi: 10.1371/journal.pone.0168348 (PMC5173368; doi:10.1371/journal.pone.0168348)
Supplement: S1 Dataset — (ZIP) [file pone.0168348.s002.zip › Taxa/Serra Norte/SN_2013/N4WS/Lista N4WS-79.pdf]

CAVIDADE N4WS-0079

Classe	Ordem	Fam/Outros	Gên/Outros	Espécie	S-U	U-S	Zon.
Arachnida	Acari	(Oribatida)		sp.1		X	
Arachnida	Acari	(Parasitengonina)		sp.3		X	
Arachnida	Acari	Argasidae	*Ornithodoros*	sp.1	X	X	E/P
Arachnida	Acari	Macrochelidae		sp.3	X		P
Arachnida	Acari	Opilioacaridae	*Neoacarus*	sp.1		X	
Arachnida	Acari	Rhagidiidae		sp.1		X	P
Arachnida	Amblypygi	Charinidae	*Charinus*	jovem	2		P
Arachnida	Amblypygi	Phrynidae	*Heterophrynus*	*longicornis*	1	1	P
Arachnida	Araneae	Ctenidae		jovem	1		E
Arachnida	Araneae	Filistatidae		jovem		X	E
Arachnida	Araneae	Ochyroceratidae		jovem	X	X	P
Arachnida	Araneae	Oonopidae	*Scaphiella*	sp.1		X	P
Arachnida	Araneae	Oonopidae		sp.1	X		P
Arachnida	Araneae	Pholcidae	*Leptopholcus*	sp.1		X	P
Arachnida	Araneae	Pholcidae	*Mesabolivar*	*eberhard*	X		P
Arachnida	Araneae	Pholcidae	*Mesabolivar*	sp.1	X		P
Arachnida	Araneae	Prodidomidae		jovem	X		P
Arachnida	Araneae	Salticidae		jovem	X		E
Arachnida	Araneae	Scytodidae	*Scytodes*	*eleonorae*	3	1	P
Arachnida	Araneae	Theridiidae	*Achaearanea*	sp.1	X	X	P
Arachnida	Araneae	Theridiosomatidae	*Plato*	sp.1	X		P
Arachnida	Pseudoscorpiones	Chernetidae		sp.1	X		P
Chilopoda	Scolopendromorpha	Cryptopidae	*Cryptops*	sp.1	1		P
Chilopoda	Scutigeromorpha	Pselliodidae		jovem		1	P
Diplopoda	Polydesmida			jovem		X	E
Entognatha	Collembola	Cyphoderidae		sp.1	X		P
Entognatha	Collembola	Entomobryidae		sp.6		X	E
Entognatha	Collembola	Paronellidae		sp.4	X		P
Entognatha	Diplura	Campodeidae		sp.1	X	X	E/P
Entognatha	Diplura	Projapygidae		sp.1		X	E
Insecta	Blattodea	Blaberidae		jovem	3	3	E/P
Insecta	Blattodea	Polyphagidae		jovem	2	1	E
Insecta	Coleoptera	Carabidae		jovem	X		P
Insecta	Coleoptera	Scydmaenidae		sp.3	X		P
Insecta	Coleoptera	Staphylinidae	Pselaphinae	sp.5	X	X	P
Insecta	Coleoptera	Tenebrionidae		jovem	X		P
Insecta	Diptera	Cecidomyiidae	Cecidomyiinae	sp.	X		P
Insecta	Diptera	Ceratopogonidae		jovem	X		P
Insecta	Diptera	Chloropidae		sp.		X	E
Insecta	Diptera	Dolichopodidae		sp.	X		E
Insecta	Diptera	Muscidae		jovem	X		P
Insecta	Diptera	Phoridae	Metopininae	sp.	X		P
Insecta	Diptera	Psychodidae	*Sciopemyia*	*sordellii*	X	X	E/P
Insecta	Heteroptera	Schizopteridae	Schizopterinae	sp.1		X	E/P
Insecta	Homoptera	Cixiidae		sp.1	X	X	P
Insecta	Homoptera	Cixiidae		sp.3	X		E
Insecta	Hymenoptera	Braconidae		sp.1	X		P
Insecta	Hymenoptera	Formicidae	*Crematogaster*	sp.3		X	P
Insecta	Hymenoptera	Formicidae	*Nylanderia*	sp.3		X	E

Insecta	Hymenoptera	Formicidae	*Solenopsis*	sp.1	X		P
Insecta	Hymenoptera	Formicidae	*Solenopsis*	sp.2		X	P
Insecta	Isoptera	Termitidae	*Convexitermes*	sp.	X	X	P
Insecta	Lepidoptera	Noctuoidea		sp.1		26	E
Insecta	Lepidoptera	Noctuoidea		sp.4	X		P
Insecta	Lepidoptera	Tineoidea		sp.4		X	E
Insecta	Neuroptera	Myrmeleontidae		jovem	1	1	E
Insecta	Orthoptera	Phalangopsidae	*Paraclodes*	sp.	5	6	P
Insecta	Psocoptera	Psyllipsocidae	*Psyllipsocus*	sp.1		X	E
Gastropoda	Pulmonata	Bulimulidae	*Naesiotus*	sp.		X	P
Gastropoda	Pulmonata	Systrophiidae	*Happia*	sp.	X	X	E/P
Malacostraca	Isopoda	Armadillidae		sp.1	X		E
Symphyla		Scutigerellidae	*Hanseniella*	sp.1	X	X	E/P
Amphibia	Anura	Leptodactylidae	*Leptodactylus*	cf. *vastus*		1	E
Amphibia	Anura	Strabomantidae	*Pristimantis*	cf. *fenestratus*	3	1	E/P
Reptilia	Squamata	Gekkonidae	*Thecadactylus*	sp.		1	P
